# ﻿New species of the dancing semislug *Cryptosemelus* Collinge, 1902 (Eupulmonata, Ariophantidae) from Loei Province, northeastern Thailand with a key to genera of mainland Southeast Asian semislugs and a key to species of the genus

**DOI:** 10.3897/zookeys.1163.103650

**Published:** 2023-05-19

**Authors:** Sakboworn Tumpeesuwan, Kitti Tanmuangpak, Chanidaporn Tumpeesuwan

**Affiliations:** 1 Department of Biology, Faculty of Science, Mahasarakham University, Kantharawichai District, Maha Sarakham 44150, Thailand; 2 Palaeontological Research and Education Centre, Mahasarakham University, Kantharawichai District, Maha Sarakham 44150, Thailand; 3 Program of Biology, Department of Science, Faculty of Science and Technology, Loei Rajabhat University, Loei 42000, Thailand

**Keywords:** Genital system, karst topography, limestone hill, mantle extensions, protective behavior, shell lobes

## Abstract

In this study, we describe a new dancing semislug from a limestone hill area in northeastern Thailand. *Cryptosemelusniger***sp. nov.** differs from the three recognized congener species from western and southern Thailand, due to differences in their body and shell lobes coloration, appearance of penial caecum, shape and surface texture of penis and epiphallus, and radula formula and morphology.

## ﻿Introduction

*Cryptosemelus* Collinge, 1902 is a member of family Ariophantidae. Its common characters comprise a shell with reduced number of whorls, body with well-developed mantle extensions, tail without caudal horn and genitalia without flagellum and amatorial organ. This genus differs from other long-tail semislug genera described from the Malay Peninsula. It differs from *Apoparmarion* due to more shell whorls and the absence of a caudal horn, flagellum and dart apparatus; and, it differs from *Paraparmarion* by the presence of both right and left shell lobes, whereas, the latter genus presents only a right shell lobe (Collinge 1902; Pholyotha et al. 2021a) (Table 1).

**Table 1.** Comparison of morphological characters among three semislug genera, *Cryptosemelus*, *Apoparmarion* and *Paraparmarion* (data from Collinge 1902; Schileyko 2003; Pholyotha et al. 2021a).

Characters	*Cryptosemelus*	*Apoparmarion*	*Paraparmarion*
Number of shell whorls	3–4	2	3–4
Left shell lobe	Present	Present	Absent
Caudal horn	Absent	Present	Absent
Flagellum	Absent	Present	No information
Dart apparatus	Absent	Present	No information

*Cryptosemelus* has been referred to as a ‘dancing semislug’ because of its dance-like movement that it makes when it is disturbed or attacked (Collinge 1902). This protective behavior has also been reported in other species, including *Laocaiasimori* Dedov & Schneppat, 2019 in Dedov et al. (2019), *Cryptausteniaaltatorial* Wiktor, 2002 and *Cryptausteniamirabilis* Wiktor, 2002, *Muangnuaarborea* Tumpeesuwan & Tumpeesuwan, 2019; *Cryptosemelusgracilis* Collinge, 1902, *C.betarmon* Pholyotha, 2021 and *C.tigrinus* Pholyotha, 2021 in Pholyotha et al. (2021a), *Ibycus* spp. and *Helicarion* spp. (Junn Kitt Foon, pers. comm.)

According to a recent study by Pholyotha et al. (2021a), the type species of the genus, *Cryptosemelusgracilis*, and the recently named species *C.betarmon* and *C.tigrinus* were studied and described from their genital anatomy and radula morphology. These three species are characterized by differences in the anatomical details of their penis, epiphallus and spermatophore. We discovered and examined this lovely semislug from a limestone hill in Loei Province, Thailand, and it possesses distinct characters of external shell morphology, mantle lobes coloration, radula and genital organs. Thus, we describe it here as a new species of *Cryptosemelus*.

## ﻿Material and methods

**Figure 1.**
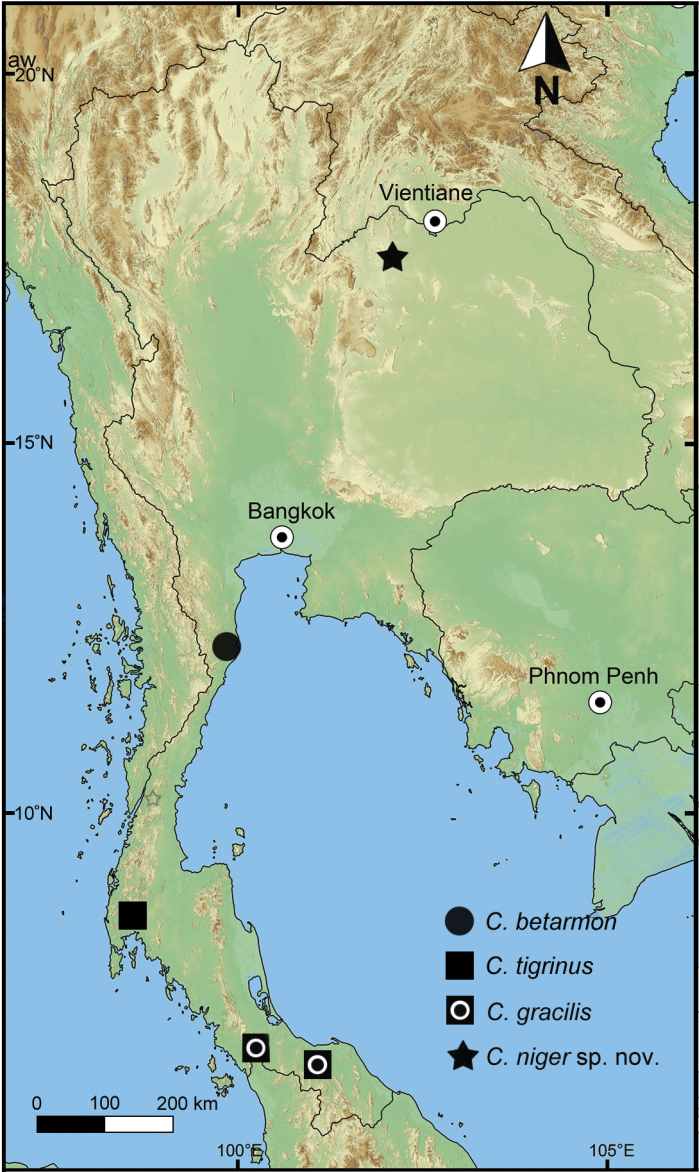
Map of type locality of *Cryptosemelusniger* S. Tumpeesuwan & C. Tumpeesuwan, sp. nov.

**Figure 2.**
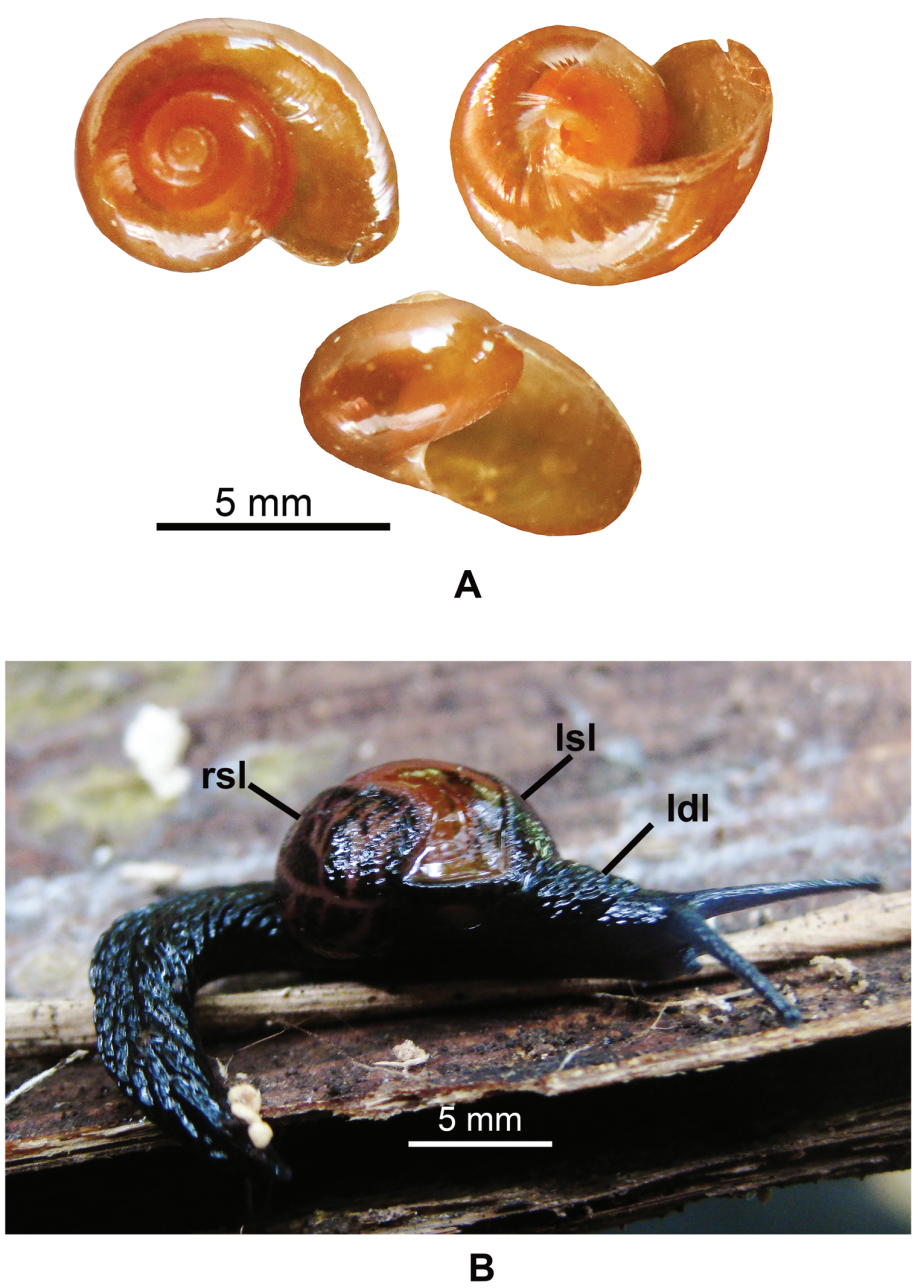
*Cryptosemelusniger* S. Tumpeesuwan & C. Tumpeesuwan, sp. nov. Photograph by Kitti Tanmuangpak **A** shell morphology of holotype NHMSU-00054 **B** living snail.

**Figure 3.**
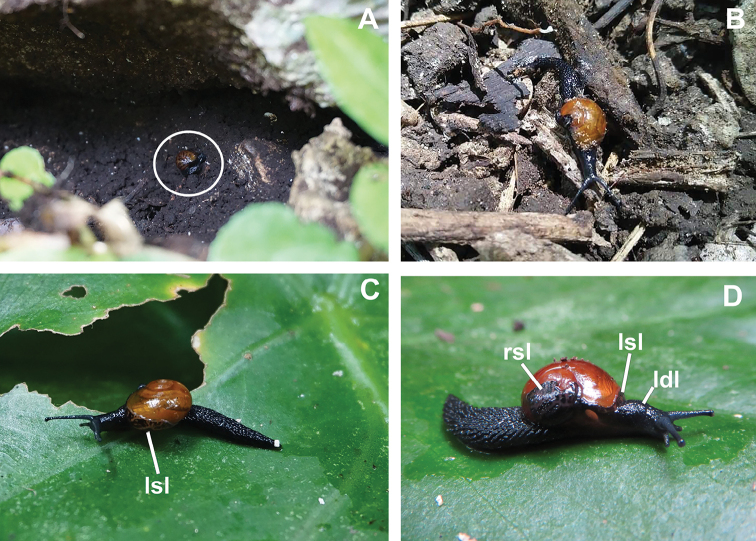
*Cryptosemelusniger* S. Tumpeesuwan & C. Tumpeesuwan, sp. nov. Living snail in natural habitats. Photograph by Chanidaporn Tumpeesuwan 12 December 2018 **A***C.niger* crawling on soil in limestone crevice (white circle) **B** close-up view in living position **C, D** semislug was moved to green leaf for taking photo **C** left side represents left shell lobe (lsl) **D** right side represents right shell lobe (rsl), left shell lobes (lsl), and left dorsal lobe (ldl).

Sixty specimens were collected from November to December 2012, June to September 2013, and 12^th^ October 2018 in the Phu Pha Lom limestone area (17°33'62"N, 101°52'31"E), elevation about 380–390 m above mean sea level, in the Mueang Loei District, Loei Province, northeastern Thailand (Fig. 1). The specimens were composed of fifty empty shells and 10 living specimens, which were collected from both leaf litter and the ground surface. The living specimens were photographed in their natural habitat (Figs 2B, 3), and then euthanized and preserved in 70 (v/v) ethanol for morphological and anatomical studies. Specimens were classified and identified from the literature, such as Collinge (1902), Blanford and Godwin-Austen (1908), Solem (1966), Schileyko (2003) and Pholyotha et al. (2021a). For the descriptive study, adult shells were measured for size using a vernier caliper and the number of whorls were counted. Photomicrographs were taken using a scanning electron microscope (JEOL, JSM-6460 LV) housed at the Central Laboratory, Faculty of Science, Mahasarakham University, Thailand. Eight specimens were dissected and examined under a stereoscopic light microscope.

Descriptions of the new species herein are attributed to the first and the third authors, as indicated below. Type specimens and other voucher specimens were deposited in the Natural History Museum of Mahasakham University, Maha Sarakham, Thailand (NHMSU).

The abbreviations used were as defined by Blanford and Godwin-Austen (1908), Solem (1966), Pholyotha et al. (2020) and Pholyotha et al. (2021a, b): **ag**, albumen gland; **at**, atrium; **e1**, portion of epiphallus nearer to penis; **e2**, portion of epiphallus nearer to retractor muscle; **fo**, free oviduct; **gd**, gametolytic duct; **gs**, gametolytic sac; **hd**, hermaphroditic duct; **ldl**, left dorsal lobe; **lsl**, left shell lobe; **ovt**, ovotestes; **p**, penis; **prm**, penial retractor muscle; **pro**, prostate gland; **rsl**, right shell lobe; **v**, vagina; **vd**, vas deferens; **ut**, uterus.

For the description of the genital system; ‘proximal’ refers to the region closest to the genital opening and ‘distal’ refers to the region outermost from the genital opening.

## ﻿Results

### ﻿Systematic description

**Superfamily Helicarionoidea Bourguignat, 1877**

**Family Ariophantidae Godwin-Austen, 1883**

**Subfamily Ostracolethinae Simroth, 1901**

#### ﻿Genus Cryptosemelus

Taxon classificationAnimaliaStylommatophoraAriophantidae

Collinge, 1902

63B90898-BAD6-5E90-8C70-FF52E0385E56

Cryptosemelus Collinge, 1902: 76. Blanford and Godwin-Austen 1908: 180. Thiele 1931: 640. Zilch 1959: 326. Vaught 1989: 97. Schileyko 2003: 1332. Bank 2017: 53. Inkhavilay et al. 2019: 75. Pholyotha et al. 2021a: 43–65.

##### Type species.

*Cryptosemelusgracilis* Collinge, 1902. *Cryptosemelus* has a reduced shell of three to four whorls, well-developed mantle extensions with two dorsal lobes and right shell lobe covering the apex and larger than the left shell lobe. Caudal horn absent. Genital system without flagellum and dart apparatus.

#### ﻿ Cryptosemelus niger

Taxon classificationAnimaliaStylommatophoraAriophantidae

S. Tumpeesuwan & C. Tumpeesuwan sp. nov.

2D76BE33-3DC0-553E-8CB9-1F12CE5A4AF2

https://zoobank.org/FA7C7967-4A62-4A6E-992C-4BE2E6DC9B36

Figs 1 , 2 , 3 , 4

Cryptaustenia sp. Tanmuangpak 2016: 109–110.
Cryptaustenia sp. Chimsaeng 2019: 41–42.

##### Type material.

***Holotype*.**NHMSU-00054 (Fig. 3). Phu Pha Lom limestone area, Mueang Loei District, Loei Province, northeastern Thailand, coll. Kitti Tanmuangpak, Nov. 2012. ***Paratypes*.**NHMSU-00055. Same locality and same date as holotype.

##### Type locality.

Phu Pha Lom limestone area, Mueang Loei District, Loei Province, Thailand.

##### Diagnosis.

**Figure 4.**
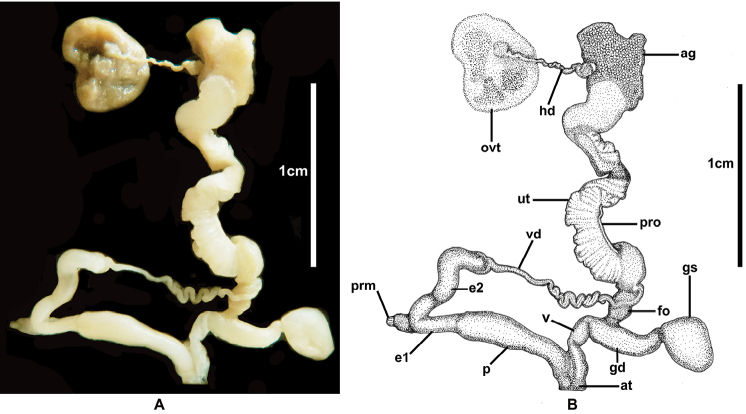
Genital system of *Cryptosemelusniger* S. Tumpeesuwan & C. Tumpeesuwan, sp. nov. (paratype NHMSU-00055) **A** photograph and **B** drawing by Kitti Tanmuangpak.

**Figure 5.**
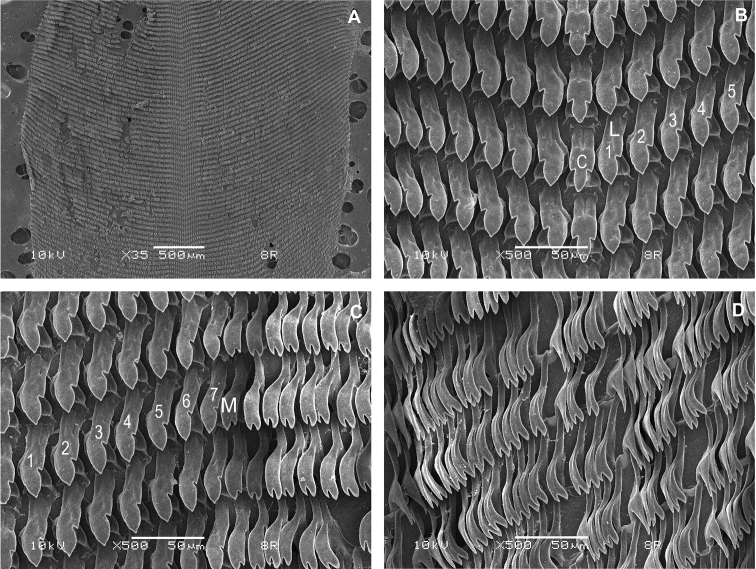
Radula morphology of *Cryptosemelusniger* S. Tumpeesuwan & C. Tumpeesuwan, sp. nov. (paratype NHMSU-00055) **A** radula plate, teeth rows arranged in wide V-shape **B** close-up view of middle part of radula **C** close-up view of right side of radula **D** close-up view of right side of radula showing marginal teeth. Central teeth indicated by ‘C’; lateral teeth indicated by ‘L’; marginal teeth indicated by ‘M’.

Animal with blackish body, shell lobes with blackish reticulated skin (Figs 2, 3). Genitalia with very short cylindrical vagina, smooth elongated cylindrical epiphallus, without penial caecum (Fig. 4). Radula with bicuspid lateral teeth (Fig. 5B, C).

##### Description.

(empty shells = 8, living specimen = 4) ***Shell*** (Fig. 2A). Shell globose, small size (shell height 6.03 ± 0.71.00 mm, shell width 9.72 ± 1.32 mm), shell imperforate, thin, smooth, dark brown color; transparence, aperture large (aperture height 5.28 ± 0.68 mm and aperture width 5.71 ± 0.81 mm).

***Genital system*** (*N* = 3) (Fig. 4). Atrium (at) short. Penis rather long and cylindrical, with thin penial sheath covering entire penis. Penial retractor muscle (prm) present, short, thin and attached at junction of e1 and e2. Epiphallus (e1+e2) length is slightly equal to penis length, surface smooth, e1 cylindrical and gradually smaller in diameter, e2 cylindrical and larger than e1. Flagellum absent. Vas deferens long. Vagina is shorter than penis, cylindrical. Gametolytic duct (gd) thickened at base, gametolytic sac (gs) swollen gland at distal end. Free oviduct (fo) is shorter than vagina. Uterus and prostate gland long and stout.

***Radula*** (*N* = 3) (Fig. 5). Teeth arranged in a wide V-shape with half row formula: 1-7-70+teeth. Central teeth symmetric tricuspid. Lateral teeth and marginal teeth gradually changing from broad to narrow bicuspid.

***External appearance*** (Figs 2B, 3). Living semislug with reticulated skin, blackish to dark body marked by conspicuous grooves running downward. Four mantle extensions well developed and same color body. Shell lobes do not cover entire shell. Caudal horn absent.

##### Etymology.

The species name “*niger*” is derived from Latin word, meaning “black” referring to blackish body and mantle extensions.

##### Distribution, habitat and behavior observations.

*Cryptosemelusniger* sp. nov. is a species restricted to the dry evergreen forest that covers the Phu Pha Lom limestone area, Loei Province, Thailand. We searched after rain and found the specimens normally hiding on the ground and in the leaf litter. When the snails were disturbed, they escaped by quickly flipping and waging their tail. Information on its natural predators is unknown, but the carnivorous land snails, *Discartemon* sp., *Oophana* sp. (Streptaxidae), and *Sinoennealoeiensis* Tanmuangpak & S. Tumpeesuwan, 2015 (Diapheridae) were found in the same locality (Tanmuangpak et al. 2012; Tanmuangpak et al. 2015; Tanmuangpak 2016).

##### Remarks.

*Cryptosemelusniger* sp. nov. differ from other *Cryptosemelus* species by its black body and mantle lobes. The shell seems to have a more rapidly descending whorl than in *C.gracilis* and *C.betarmon*, but is similar to *C.tigrinus*. *Cryptosemelusgracilis* also lack a penial caecum but differs in the undulated surface patch on the proximal part of e2. The epiphallus and penis are cylindrical without a caecum, diverticulum, and granulate surface. The radula of the new species differs from all *Cryptosemelus* in having bicuspid lateral teeth, whereas other species have tricuspid lateral teeth (Table 2, Figs 2A, B, 3A–D, 4, 5B–D).

**Table 2.** Comparison of shell, genital system and radula of *Cryptosemelus* spp. (data for *C.gracilis*, *C.betarmon* and *C.tigrinus* based on Pholyotha et al. 2021a).

Characters	*C.gracilis*	*C.betarmon*	*C.tigrinus*	*C.niger* sp. nov.
**Shell**:				
Shape	Less, globose	Depressed, subglobose	Globose	Globose
Shell width	Up to 6.6 mm	Up to 7.4 mm	Up to 10.7 mm	Up to 11.04 mm
Shell height	Up to 4.2 mm	Up to 4.1 mm	Up to 7.6 mm	Up to 6.74 mm
Whorls number	3 ½ –4	3 ½ –4	4–4 ½	3–4
Shell color	Pale golden amber	Pale yellowish with olive tinge	Pale yellowish with olive tinge	Dark brown transparent
**Living snails**:				
Shell lobes coloration	Monochrome blue-gray to blackish	Monochrome pale to dark-grayish	Pale yellowish-orange banded	Blackish reticulated skin
**Radula**:				
Radula formula	1-(19-20)-38	1-(27-28)-37	1-(38-39)-44	1-7-70+
Central tooth	Tricuspid	Tricuspid	Tricuspid	Tricuspid
Lateral teeth	Tricuspid	Tricuspid	Tricuspid	Bicuspid
Marginal teeth	Tricuspid	Bicuspid	Bicuspid	Bicuspid
**Genital system**:				
Epiphallus e2: Proximal part	Enlarged with irregularly undulated surface patch	Enlarged and with undulated surface	Cylindrical smooth surface	Long cylindrical with smooth surface
Epiphallus e2: Distal part	Smooth surface	Cylindrical and gradually tapering to distal end, smooth surface	Cylindrical with prominently granulated surface	Long cylindrical with smooth surface
Penial caecum	Absent	Present	Present	Absent
Vagina	Larger and shorter than penis length	Smaller and shorter than half of penis length	Long slender and longer than half of penis length	Smaller and shorter than penis length

## ﻿Discussion

The absence of a caudal horn is the unique character shared between *Cryptosemelus* and *Paraparmarion* (Collinge 1902; Blanford and Godwin-Austen 1908; Solem 1966; Schileyko 2002, 2003; Pholyotha et al. 2021a). *Cryptosemelus* differs from *Paraparmarion* in that the left shell lobe is well developed, whereas it is missing in *Paraparmarion* (Collinge 1902; Pholyotha et al. 2021a).

All previously described species of *Cryptosemelus* show no epiphallic caecum, flagellum, and dart apparatus. Shell lobes coloration, appearance of the penial caecum, shape and surface sculpture of the epiphallus, and radula morphology are considered as taxonomically informative and these can be used to distinguish the new species from all recognized *Cryptosemelus* species.

We have improved the key to the genera of mainland Southeast Asian slug-like semislugs provided by Tumpeesuwan and Tumpeesuwan (2019b) for identifying slug-like semislugs in mainland Southeast Asia and provide a key to species of genus *Cryptosemelus* below.

### ﻿Key to genera of mainland Southeast Asian slug-like semislugs

**Table:** 

1	Finger nail or triangular-shaped shell, always covered by shell lobes	**2**
–	Ear-shape, subglobose, or globose-shaped shell, frequently covered by shell lobe	**3**
2	Finger nail-shaped shell; gametolytic sac long, cylindrical tube	***Muangnua***
–	Triangular-shaped shell; gametolytic sac stalk, short and stout or moderately long and slender	***Laocaia***
3	Ear-shape shell; caudal horn present; dart apparatus and flagellum present	**4**
–	Subglobose; or globose-shaped shell; caudal horn absent; dart apparatus and flagellum absent or no information	**5**
4	Penis length shorter than half of dart apparatus length	***Parmarion***
–	Penis length longer than dart apparatus length	***Apoparmarion***
5	Left shell lobe present only; dart apparatus no information	***Paraparmarion***
–	Both shell lobes present, dart apparatus absent	***Cryptosemelus***

### ﻿Key to species of genus *Cryptosemelus*

**Table:** 

1	Shell lobes monochrome	**2**
–	Shell lobes with black reticulated stripes on pale colour	**3**
2	Shell globose; body blue-gray; penial caecum absent; vagina large cylindrical	***C.gracilis***
–	Shell depressed subglobose; body grayish, penial caecum present; vagina cylindrical	***C.betarmon***
3	Body color brownish; vagina long slender; penial caecum present; distal part of epiphallus with prominently granulated surface	***C.tigrinus***
–	Body color dark brown to blackish; vagina short; penial caecum absent; epiphallus with smooth surface	***C.niger* sp. nov.**

Since 2007, an intensive survey on land snail diversity in limestone and non-limestone hills in northeastern Thailand has been continuously conducted and published (Tumpeesuwan 2007; Tumpeesuwan and Tumpeesuwan 2010a, 2010b; Srihata et al. 2010; Tanmuangpak et al. 2012; [Bibr B8][Bibr B35]; Tanmuangpak 2016; Sasang 2019; Nahok 2020). In total, 16 species have been described as new to science, comprising 11 species from limestone hills, four species from sandstone hills, and one species from volcanic hills (Tumpeesuwan and Tumpeesuwan 2014, 2017, 2019a, 2019b; Tanmuangpak et al. 2015, 2017; Nahok et al. 2020, 2021a, 2021b; Deeprom et al. 2022; Tanmuangpak and Dumrongrojwattana 2022; Tongkerd et al. 2023). Future studies on the malacofauna of the northeastern part of Thailand require more surveys in overlooked and isolated natural areas.

## Supplementary Material

XML Treatment for Cryptosemelus

XML Treatment for Cryptosemelus niger
